# High specificity THz metamaterial-based biosensor for label-free transcription factor detection in melanoma diagnostics

**DOI:** 10.1038/s41598-023-46876-5

**Published:** 2023-11-24

**Authors:** Merle Richter, Yannik Loth, Anna Katharina Wigger, Daniela Nordhoff, Nicole Rachinger, Christian Weisenstein, Anja Katrin Bosserhoff, Peter Haring Bolívar

**Affiliations:** 1 High Frequency and Quantum Electronics, University of Siegen, 57076 Siegen, Germany; 2 Biochemistry and Molecular Medicine, Friedrich-Alexander-University Erlangen-Nürnberg (FAU), 91054 Erlangen, Germany

**Keywords:** Terahertz optics, Melanoma, Metamaterials

## Abstract

In this work, we present a promising diagnostic tool for melanoma diagnosis. With the proposed terahertz biosensor, it was possible to selectively and sensitively detect the early growth response protein 2, a transcription factor with an increased activity in melanoma cells, from a complex sample of cellular proteins. Fundamentally, the sensor belongs to the frequency selective surface type metamaterials and consists of a two-dimensional array of asymmetrically, doubly split ring resonator unit cells. The single elements are slits in a metallic layer and are complemented by an undercut etch. This allows a selective functionalization of the active area of the sensor and increases the sensitivity towards the target analyte. Hereby, specific detection of a defined transcription factor is feasible.

## Introduction

Melanoma, a malignant tumor emerging from melanocytes, is the deadliest type of skin cancer^1^. However, if diagnosed early, the survival rates increase drastically, thereby emphasizing the need for reliable, specific, and sensitive detection methods. Current techniques often depend on visual observations by a physician as well as immunohistochemistry to determine whether a lesion is of melanocytic origin and to investigate proteins as tumor-biomarker^1^. Here, recent approaches focus on the detection of clinical or prognostic markers as well as new types of biomarkers based on epigenetic dysfunction in melanoma cells. The term epigenetic dysfunction refers to alterations of gene regulation and other epigenetic processes in tumor cells. Some of these deviations, for instance in transcription factor activity, can be used as biomarkers and thereby facilitate novel pathways for cancer diagnosis. One example of epigenetic dysregulation in melanoma is the NAB2/EGR system (NAB2: NGFI-A binding protein 2, NGFI-A: nerve growth factor-induced protein A, EGR: early growth response protein) which is associated with several malignancies, especially in melanoma^2,3^. One consequence of the dysregulation is the overexpression of EGR2, a zinc finger transcription factor. Due to the resulting increased activity of EGR2 in melanoma, it is used as a biomarker within this work.

On account of its function as transcription factor, EGR2 binds selectively to a specific double stranded DNA (dsDNA) sequence. The single DNA strands of this sequence (ssDNA) can be synthesized individually and one strand can be modified on one end with a thiol functional group (tssDNA) to be immobilized on a gold interface.

In the field of disease and more precisely cancer diagnostics, terahertz (THz) techniques, especially biosensors have gained attention in the past years^4^. The THz frequency band is located between microwaves and infrared radiation in the electromagnetic spectrum, covering a frequency range starting from 100 GHz to 10 THz, that corresponds to a wavelength of 3 mm to 30 $\upmu$m. Application examples cover many disciplines, such as imaging or spectroscopy^5–9^. Another interesting field of research which is increasingly gaining importance is the development of biomedical applications that connect terahertz measurement methods with biology and medicine^10–12^. This approach originates from the spectral behavior of biomolecules, including intra- and intermolecular vibration or torsion modes in the THz frequency range^13,14^ making them suitable for detection with and analysis by THz techniques. Previous results have shown successful analysis of living or devitalised tissue^15–19^, RNA and DNA^20–23^, single cells and microorganisms^24,25^ as well as proteins^26–33^, demonstrating the great potential of THz radiation in the field of biomedicine. However, as the analyte size (typically $< 100$ nm) is relatively small compared to the wavelength of terahertz radiation (300 $\upmu$m at 1 THz), a straight-forward spectroscopic approach does not provide sufficient sensitivity. This mismatch can be overcome by developing suitable THz biosensors based on a special category of metamaterials, so-called frequency selective surfaces (FSS), that allow the detection of target biomolecules with high sensitivity. Despite of such an advantageous *sensitivity*, many publications only show non-selective detection or fail to prove the *selectivity* of their sensing principle, as critically pointed out in a recent review by Markelz et al^10^. Such biosensors are immediately less attractive for real life science or medical applications. A few recent publications are starting to demonstrate selectivity by allowing to distinguish within a selection of purified biomolecules (typically differentiating among up to five different biomolecules)^25,31,32,34^.

In this work, we present a highly selective and sensitive biosensor for melanoma diagnosis capable of specific EGR2 detection from a complex sample matrix directly derived from complete melanoma cell nuclei (containing approximately 7000 different protein species).

## Results

### Sensor design and principle

**Figure 1:**
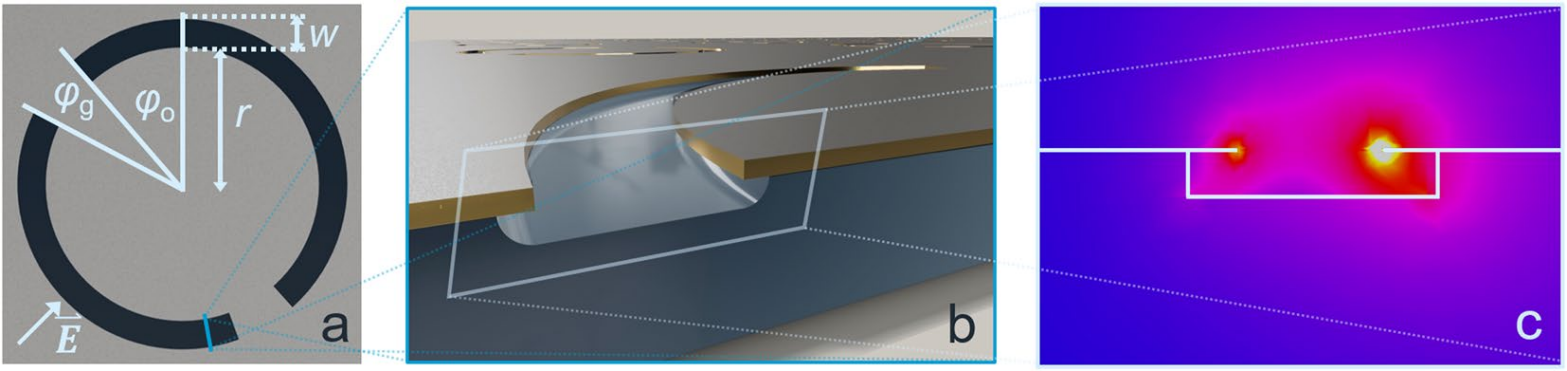
(**a**) Array element of metamaterial with design resonance frequency $f_\text{R}\,=\,600$ GHz, inner radius $r\,=\,46\,\upmu$m, arc width $w\,=\,10\,\upmu$m, gap angle $\varphi _\text{g}\,=\,22^\circ$, offset angle $\varphi _\text{o}=\,42^\circ$, periodicity of the unit cell $p\,=\,202\,\upmu$m and number of elements per query field $n\,=\,145$. (**b**) Cross section of the resonator, visualizing the $3\,\upmu$m deep undercut-etch and the sensor composition: from top to bottom: chromium (10 nm), gold (200 nm), chromium (10 nm), fused silica ($500\,\upmu$m). The dimensions are not depicted to scale. (**c**) Localized electric field concentration along the cross section of the resonator element. High field concentration displayed in yellow, low field concentration displayed in blue.

All measurement results were obtained using one FSS-based sensing platform with a design resonance frequency $f_{\text{R}}$ of 600 GHz. High frequency simulations as well as fabrication details for this sensor design have already been shown earlier in a DNA analysis publication^35^. A single element of the metamaterial array is shown in Fig. 1a. It consists of two slightly differently sized slits within a chromium-gold-chromium metal multilayer. The ends of the slits are bent towards each other resembling a doubly disrupted ring pattern. The transmission behavior of these two asymmetric arcs can physically be described using a model for light interaction in two photonic resonators coupled to each other^36^. According to the Babinet principle, the electric field in this slit conformation behaves identical to the magnetic field in the complementary metal rod conformation scenario and vice versa^37,38^. Taking these fundamentals into account, the frequency response upon excitation with electromagnetic radiation in the corresponding frequency range and polarization direction contains a resonance feature with a steep flank which is sensitive to dielectric changes in close vicinity. The model for this phenomenon is known as Fano resonance or dual resonance feature (DRF)^36^. In Fig. 1c, the electric field concentration is displayed for a cross section of one arc. The result reveals that the highest electric field concentration is located at the edge of the resonator element. By introducing an undercut-etch of $3\,\upmu$m into the fused silica substrate and underneath the metal layers, it is possible to selectively expose gold in the area of this high electric field concentration while the less sensitive areas remain covered by chromium as shown in Fig. 1b, c. Due to the fabrication process, the chromium remains only on the top side of the sensor or in direct contact with both fused silica and gold, while the area on the inside of the undercut-etch exposes the gold layer. This enables the selective functionalization of the interfaces located in the zone with the highest electric field concentration by thiol-based surface chemistry.

The single elements depicted in Fig. 1a are arranged in a two-dimensional array with a periodicity of $p\,=\,202\,\upmu$m. One array of 145 elements forms a query field (3 mm $\times$ 3 mm) acting as measurement spot that can repeatably and precisely be positioned in the focus of the THz measurement setup with the aid of positioning pinholes at the corners of the sensor chip. The query fields are organized in a chessboard-like pattern as can be seen in Table 1, which simplifies individual functionalization by placing a droplet of the corresponding solution on top of the query field in question.

### Specific EGR2 detection

**Table 1:**
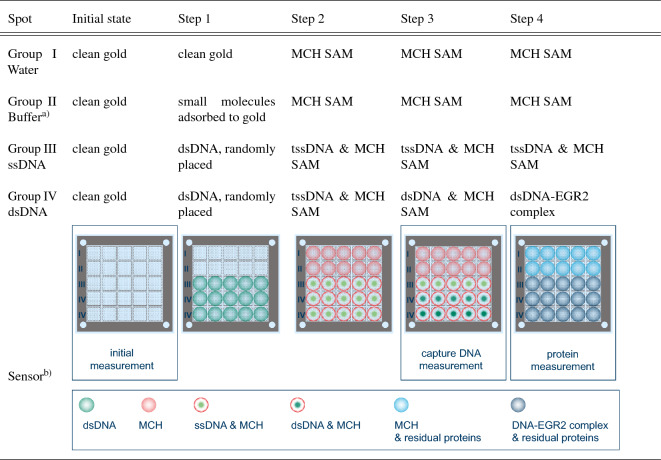
Functionalization status of the gold surfaces on each query field after the corresponding preparation step was conducted in the sample measurement.

In Step 2, all fields are treated with MCH leading to a self-assembled monolayer (SAM) formation. Randomly placed dsDNA refers to loosely adsorbed molecules in addition to thiol-bound dsDNA. The row with schematic depictions of the sensor layout and the various surface functionalization states for the corresponding query fields also indicates at which stages the measurement iterations were performed.

$^\text{a)}$ Phosphate buffer saline (PBS)

$^\text{b)}$ Depiction of entire biosensor device including 25 individually addressable query fields.

**Figure 2:**
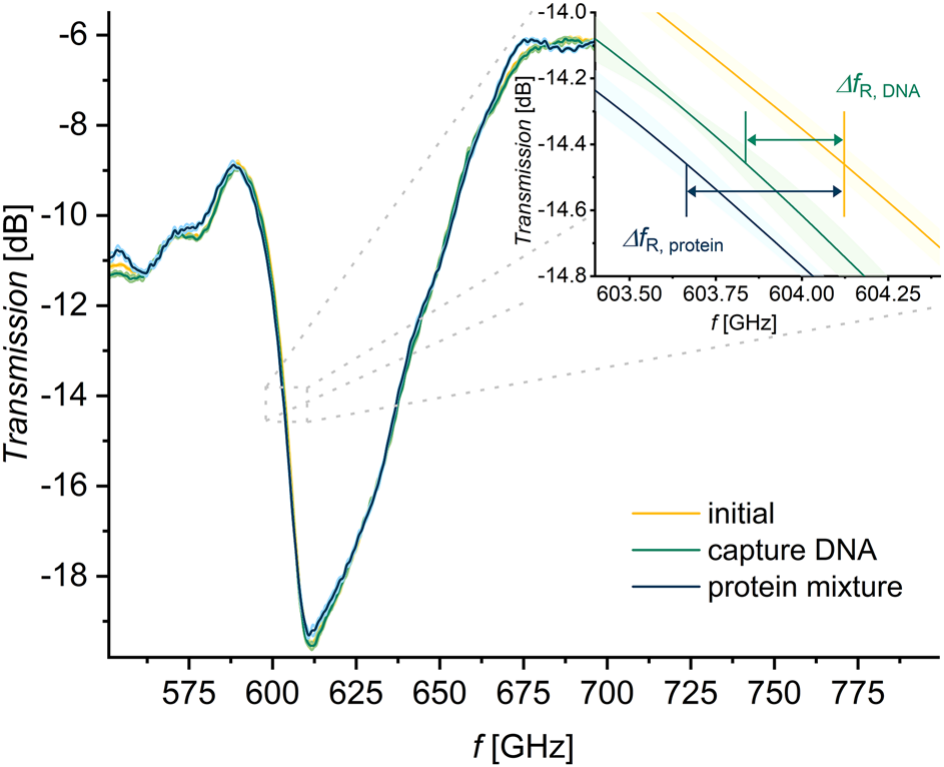
Exemplary frequency response for one measurement spot being sequentially functionalized with a biofilm here shown in logarithmic scale. Yellow plot: Clean sensor surface with exposed gold in undercut-etch area. Green plot: dsDNA was immobilized in the undercut-etch area of this query field, filling the gaps in the dsDNA biofilm with mercaptohexanol (MCH), thereby covering up the remaining exposed gold surface effectively. The term capture DNA refers to the capability of the present DNA film to selectively bind EGR2 transcription factors. Blue plot: In the measured spot, the sensor was treated with a solution from nucleus proteins extracted from human malignant melanoma cells. The translucent bands around the solid curves indicate the standard deviation $\sigma _{(N\,=\,5)}$ of each measurement stage. The inset illustrates how the resonance frequency shift $\Delta f_\text{R}$ is evaluated.

An example for the transmission frequency response is depicted in Fig. 2 visualizing the Fano resonance behavior between 580 GHz and 680 GHz. While Fig. 2 displays only one query field or measurement spot from group IV (dsDNA), this type of transmission spectrum series was recorded for each query field on the biosensor device. The three curves represent three sequential measurements of the same measurement spot at different stages of the surface functionalization procedure which is summarized in Table 1 and depicted in Table 1, Fig. Initial state-Step 4. The three measurement stages are indicated in the depiction in Table 1, Fig. Initial state, Step 3 and Step 4, as well. The functionalization procedure is described and visualized in the “Methods” section in more detail. The yellow plot has been recorded directly after the sensor was cleaned, while the green one has been measured after covering the gold with a biofilm consisting of dsDNA and MCH (see Table 1, step 3 in the functionalization procedure for group IV sample measurement spots), thereby altering the dielectric environment in the high electric field area of the sensor and causing a shift of the DRF towards lower frequencies. After that measurement, the sensor chip undergoes the next step in the preparation procedure (see Table 1, step 4 in the functionalization procedure for group IV sample measurement spots), leading to a further negative shift of the resonance frequency. This time, the change to a lower resonance frequency is caused by the presence of proteins from melanoma nuclei. The detailed bioanalytic procedure is described in the methods section. The small values of the resonance shifts induced by the biomolecules illustrate the need to precisely analyze the statistical validity and variances of different measurements, as illustrated in the inset of Fig. 2.

**Figure 3:**
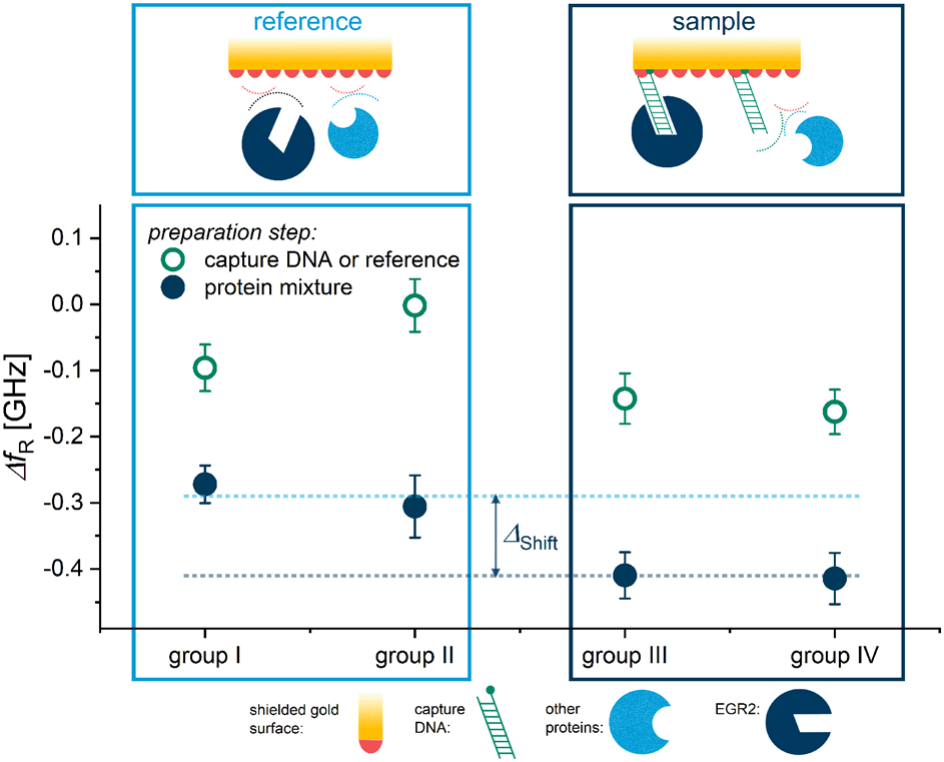
Resonance shifts for different measurement spot groups in the sample measurement series, using the sample measurement treatment. Reference spots were treated either with water (Group I) or PBS (Group II) resulting in a complete shielding of the gold surface. Sample measurement spots contain capture DNA which refers to either a ssDNA film (Group III) or a dsDNA film (Group IV), allowing a selective immobilization of EGR2 transcription factors.

A corresponding series of transmission spectra comparable to the exemplary one in Fig. 2 was generated for each query field with the individual spectra being recorded at different stages of the sensor preparation procedure illustrated in Table 1. The fields can be divided into four groups (groups I–IV) inside of which they were treated equally, and an average frequency shift is determined for each group and measurement iteration. The resulting frequency shifts for the entire measurement sequence, featuring the various query field groups in different functionalization stages are visualized in Fig. 3.

The sulfur atom in MCH as well as in the tssDNA forms a strong bond to gold and supersedes any loosely adsorbed molecules this way. This also removes DNA that is only physically adsorbed to the gold surface and not anchored by its thiol-functional group in Table 1, step 2 on the ssDNA and dsDNA fields. Subsequently, after denaturing the bound DNA strands in Table 1, step 2, the fields which finally carry dsDNA are hybridized again using the complementary ssDNA (Table 1, step 3). With this, the biosensor functionalization is complete. At this point, the query fields can be categorized into reference spots and sample spots which are expected to behave differently when exposed to a solution of nucleus proteins containing the transcription factor that selectively binds to the dsDNA sequence on the corresponding sample fields. In Table 1, step 4, which can be considered as the actual bioanalytic query on the functionalized biosensor, the sensor is exposed to the protein sample.

The different surface functionalization of sample spots and reference spots results in deviating resonance frequency shifts as depicted in Fig. 3. The resonance frequency shift $\Delta f_\text{R}$ is visualized as a function of the type of measurement spot. The different steps in the sensor preparation procedure are displayed with different colors and symbols as data points. The inset in Fig. 2 demonstrates that the frequency shifts are all referenced to the initial measurement of the corresponding measurement spot with a clean surface: $\Delta f_\text{R, Step 3}$ $=f_\text{R, Step 3}$ $-f_\text{R, initial}$ and $\Delta f_\text{R, Step 4}$ $=f_\text{R, Step 4}$ $-f_\text{R, initial}$.

The measurement spots are grouped into reference spots and sample spots, which is complemented by a pictogram displaying the molecular situation at the gold interface. Due to the MCH shielding, no unspecific binding of proteins can occur in these areas which prevents adsorption of almost all nucleus proteins in the sample solution. Only those proteins that can specifically bind to the prepared dsDNA sequence are able to form a complex and get immobilized. In Fig. 3, it is shown that with $\Delta f_\text{R, sample}$ $\,=\,-0.41$ GHz, the sample fields experience a substantially larger shift towards lower frequencies than the reference fields $\Delta f_\text{R, ref}$ $\,=\,-0.29$ GHz. The average difference between the frequency shifts of reference spots and sample spots after functionalization step 4 is $\Delta _\text{Shift}\,=\,120$ MHz. The error bars represent a measure for the uncertainty of the measurement and are calculated by forming the standard deviation of the five subsequently recorded differences for each spot and preparation step. The overall repeatability can statistically be evaluated by using the standard deviation over $f_\text{R}$ of equally treated spots and includes fabrication deviations between single measurement spots, environmental influences as well as differences in the biochemical functionalization process. The repeatability in this measurement sequence was determined to be $\sigma _{(\Delta \text{fR)}}\,=\,0.16$ GHz. The entire measurement sequence was conducted multiple times in the same manner and resulted consistently in identical observations of the sensing effect with similar shift amplitudes.

**Figure 4:**
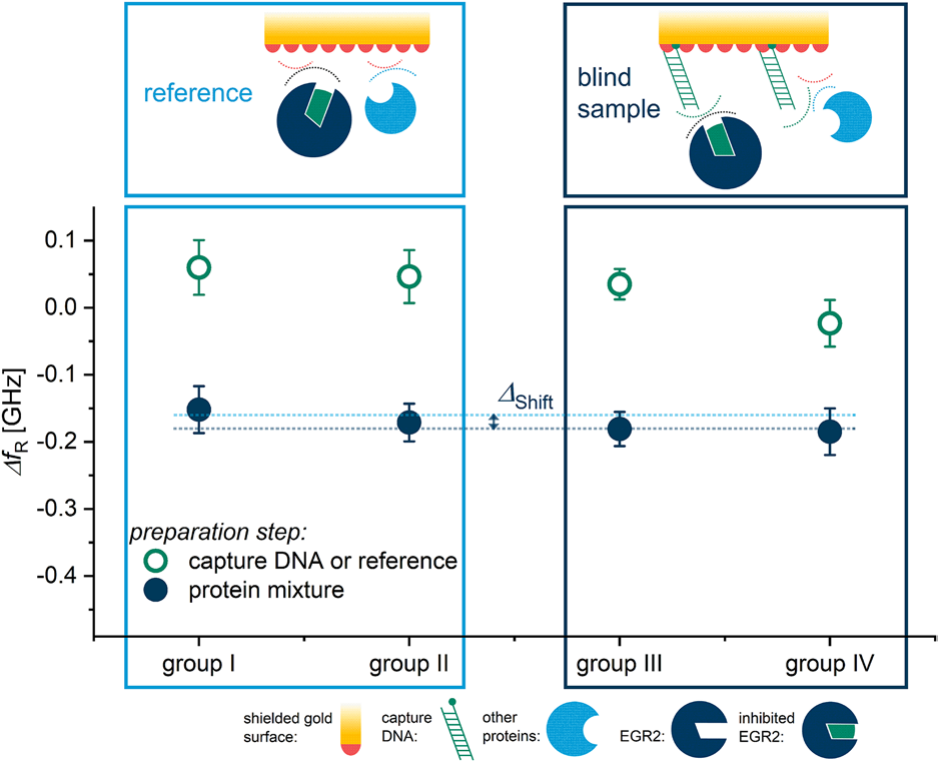
Resonance frequency shifts for different measurement spot groups in the blind sample measurement series using the blind sample treatment. Reference spots were treated either with water (Group I) or PBS (Group II) resulting in a complete shielding of the gold surface. Blind sample measurement spots contain capture DNA which refers to either a ssDNA film (Group III) or a dsDNA film (Group IV). Since EGR2 had been saturated with dsDNA $ex-situ$, no selective immobilization occurred.

A similar graph was created for the measurement results of the control or blind sample experiment and can be found in Fig. 4. The only difference between the sample experiment and the control experiment is that the core protein solution has been treated *ex-situ* with dsDNA solution II prior to its application on the biosensor. Accordingly, the protein-DNA complex forms in the blind sample solution which inhibits the EGR2 active site and therefore its immobilization on the sensor surface. The average difference between the frequency shifts of reference spots and sample spots after functionalization step 4 is $\Delta _{Shift}\,=\,20$ MHz for the control experiment.

## Discussion

The few metamaterial-based biosensors that have been reported and proved to specifically detect a tumor-marking protein species using antibody-antigen systems were tested for a couple of cross-sensitivities^31,32,34,39^. Our results show that the sensing platform proposed in this work is capable of distinguishing one sample containing an active biomarker species from another where this species is silenced. It should be emphasized that since the sample is extracted from melanoma cells, it contains on the order of 7000 different protein species that are present in the nucleus of human cells. The ratio of messenger RNA (mRNA) molecules encoding EGR2 to the total number of mRNA molecules is $3.12\,\cdot \,10^{-7}:\,1$. Despite the restricted comparability of EGR2 mRNA abundance and the resulting protein activity, this proportion allows an approximation of the EGR2 amount in melanoma cells and highlights the degree of selectivity and sensitivity of the proposed sensor. The difference of $120$ MHz between the resonance frequency shift of reference fields and dsDNA fields in Fig. 3 can only be attributed to the specific binding of EGR2 to the capture DNA. Although we did not expect a protein-DNA complex to form with ssDNA, the measurement results show that a resonance frequency shift with a similar amplitude as for double stranded capture DNA occurs. One possible explanation for this finding could be a potential temporary stem-loop formation of the single strand, leading to a double-stranded binding domain for EGR2. However, this process is not controlled and should have a smaller influence. Further investigation about the observation will be necessary to fully understand this finding. In addition to that, the control experiment displayed in Fig. 4 shows that no unspecific interaction takes place with the capture DNA, which verifies the previous hypothesis in this regard. We also observe an offset shift in resonance frequency in all reference measurement spots during the control experiment (see Fig. 4) and the sample measurement (see Fig. 3). This is most likely caused by unspecific adsorption of random nucleus proteins in the regions of the sensor where chromium or SiO${}_{2}$ are exposed. Although this unspecific adsorption can take place in a large area compared to the limited binding sites in the undercut-etch, the resulting shift in resonance frequency on the dsDNA fields in Fig. 3 can be assigned to specific binding of just one protein species, namely the transcription factor EGR2. Other works in recent literature saturate the passive sensor surface with bovine serum albumin (BSA) to prevent unspecific adsorption^29,31,32,34,39^. The concept to provide multiple measurement spots or query fields on one sensor chip allows us to record an internal reference with each measurement so that the additional saturation step with BSA is not necessary. This is possible due to the intricate double barrier design of the query fields that prevents single droplets from leaking onto neighbouring spots during the functionalization procedure as can be seen in Fig. 5b. Techniques that involve proteins instead of DNA during the functionalization procedure are more sensitive towards environmental influences including but not limited to temperature, pH and drying. Consequently, the time between functionalization and sample measurement needs to be kept as short as possible in those cases while DNA-based sensors are to some extent more durable.

## Conclusion

In this work, we demonstrated a metamaterial-based biosensor that enables the specific detection of EGR2, a biomarker that is associated with epigenetic dysfunction in melanoma. Due to its unique design and biochemical modification, it can selectively distinguish the analyte despite of its low abundance within the full complex protein matrix of the cell nucleus. One distinct advantage of the proposed biosensor is its practicability with regard to a single-photolithography fabrication process and its durability regarding the functionalization. In future research we plan to investigate the precise behavior of this and other transcription factors in the presence of their corresponding capture DNA in more detail. Moreover, an extremely important and challenging step for THz biosensing is to perform experiments in aqueous solutions in order to maintain the tertiary and if applicable quaternary structure of protein analytes during the measurement^35,40^.

## Methods

### THz measurements

**Figure 5:**
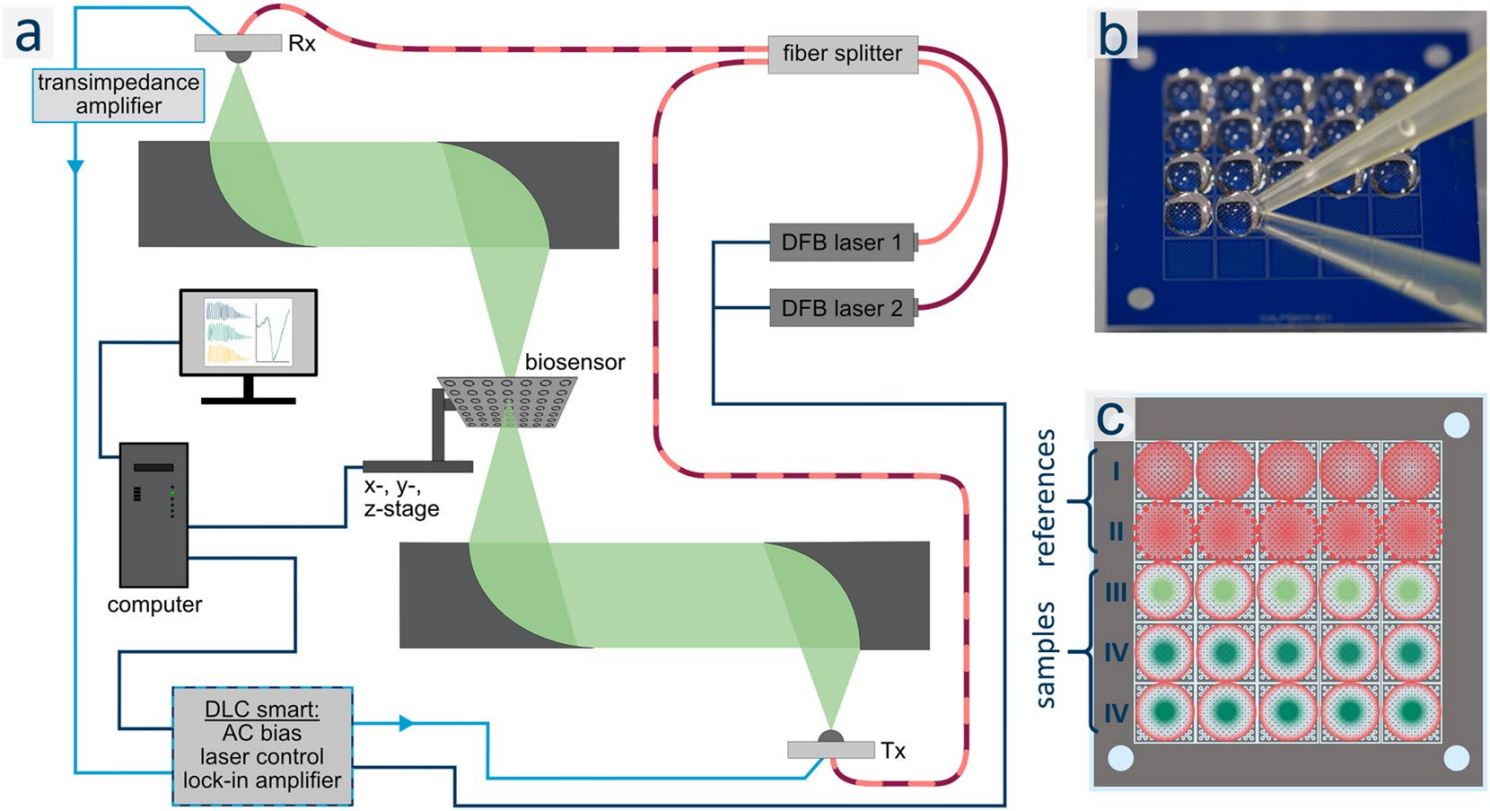
(**a**) Schematic overview of Toptica TeraScan 1550 with added optical path and data evaluation instance. The THz-beam is depicted in green, connecting fibers are displayed in light and dark red, signal lines are displayed in light blue and control lines in dark blue. Rx: receiver, DFB laser: distributed feedback laser, DLC: digital laser controller, Tx: transceiver/emitter. (**b**) Photograph of exemplary sensor preparation. (**c**) Schematic depiction of the 25 query fields and their functionalization. From top to bottom: First row, red circles: Group I-spots (water); second row, red circles with dotted outline: Group II-spots (PBS); third row, light green circles with red outline: Group III-spots (tssDNA); fourth and fifth row, dark green circles: Group IV-spots (dsDNA).

#### Measurement system

Typical terahertz time-domain systems do not provide sufficient frequency resolution to detect small frequency shifts in the MHz range. Thus, a continuous wave THz (cw-THz) spectroscopy setup operating in the frequency domain has been set up, mainly based on the TeraScan 1550 system from TOPTICA Photonics AG. This system provides a high dynamic range, a high bandwidth and a spectral resolution down to 1 MHz^41^. In order to allow a transmission measurement of the biosensor, the measurement setup was extended with off-axis parabolic mirrors accomplishing a quasi-optical transmission path with an intermediate focus. Utilizing a specially adapted vacuum chuck, the sensor is placed in the focal plane^42^.This chuck combined with motorized linear stages (MICOS LS-65) enables a precise positioning during each measurement routine. In such a way, repeatable and comparable measurements of the individual query fields can be conducted before and after the functionalization with biomolecules.

#### Measurement routine

Prior to the initial measurement routine, the THz biosensor, attached to the vacuum chuck, was calibrated with the use of alignment marks placed in the form of a small hole in the metal multilayer in each corner (Fig. 5b,c). For this purpose, all calibration holes were scanned, resulting in a Gaussian shaped terahertz intensity profile for each of them. This allows to accurately determine the center position of the calibration holes. Subsequently, the exact center position for each of the 25 query fields was calculated and utilized for each following measurement routine of the same sensor chip. The coordinate system of the chip design is thus linked to the coordinate system of the motorized linear stages, resulting in each query field being measured at the same position. For each measurement routine, the frequency was swept from 400 to 800 GHz ensuring the resonance frequency of the sensor being in the center of this frequency range. The step size in this range was set to 40 MHz and the measurement was accomplished with an integration time of 10 ms.

#### Data processing

The acquired signal from the measurement system corresponds to the photocurrent in the photo detector and is correlated to the THz electric field. Since this signal only consists of the real part, we calculated the complex data by the use of a Hilbert transform. Using an inverse fast Fourier transform (IFFT), the frequency domain data was converted into the time domain, where high frequency oscillations that do not carry information about the sample were eliminated by the application of a cosine tapered window filter. The cosine-weighted transition between the main signal inside the window and the high frequency signals outside the window avoided the introduction of artefacts in the proceeding data evaluation routine. The filtered time-domain signal was translated back to the frequency domain by applying a fast Fourier transform (FFT). The resulting signal was normalized to an air measurement being recorded directly after the sample measurements. Comparable data evaluation routines have been published before^35,43^. In order to determine the resonance frequency in a reliable manner, the signal was normalized to the local maximum $s(f{}_\text{max} )\,=\,1$ and local minimum $s(f{}_\text{min} )\,=\,0$ of the DRF and $f_\text{R}$ was defined by $s(f_\text{R})\,=\,0.5$. This parameter was found to be sensitive towards dielectric load on the sensor surface, yet being more precise than the frequency at the local minimum $s(f{}_\text{min} )$^35^. Resonance frequency shifts $\Delta f_\text{R}$ were calculated by subtracting the resonance frequency determined in the initial measurement from the one performed after step 3 or step 4 from the functionalization procedure, respectively. To obtain statistically relevant data, each spectrum was recorded five times and the arithmetic mean as well as the standard deviation of the resonance frequency shifts were calculated from the five subsequent measurements. The error bars in Figs. 3 and 4 thus indicate a measure of the uncertainty of each data point. The repeatability of the entire process (including the surface chemistry, the biochemical reactions, stage precision, environmental influences, etc.), can be judged by generating the standard deviation of the different measurement spots/query fields that were treated equally during the functionalization procedure.

### Sample preparation

Whenever water is mentioned in this work, this refers to deionized water with a conductivity $C\,<\,0.056\,\upmu$S/cm which was filtered with a pore size of $0.2\,\upmu$m.

#### Buffer solutions

(*) Buffer I: 2-[4-(2-hydroxyethyl)piperazin-1-yl]ethane-1-sulfonic acid (HEPES, $10$ mM), KCl ($10$ mM), 2,2$^{\prime }$,2$^{\prime \prime }$,2$^{\prime \prime \prime }$-(ethane-1,2-diyldinitrilo)tetraacetic acid (EDTA, $0.1$ mM), 3,12-bis(carboxymethyl)-6,9-dioxa-3,12-diazatetradecane-1,14-dioic acid (EGTA, $0.1$ mM), (2S,3S)-1,4-bis(sulfanyl)butane-2,3-diol (DTT, 1 : 1000 $1$ M), pH$\,=\,7.9$

(**) Buffer II: HEPES ($20$ mM), NaCl ($0.4$ M) EDTA ($1$ mM); EGTA ($1$ mM); DTT (1 : 1000 $1$ M), pH$\,=\,7.9$

(***) Buffer III: HEPES ($2.5$ mM), MgCl${}_{2}$ ($2.5$ mM), KCl ($25$ mM), EDTA ($0.1$ mM), DTT ( $2.5$ mM), pH$\,=\,7.9$

(****) Buffer IV: NaCl ($1.37$ M), NaH${}_{2}$PO${}_{4}$ $\,\cdot$ H${}_{2}$O ($0.1$ M), KCl ($27$ mM), KH${}_{2}$PO${}_{4}$ ($18$ mM), pH$\,=\,7.0$

#### DNA oligomer solutions

All DNA oligomers were sourced off of Eurofins Genomics, Ebersberg, Germany.

*dsDNA solution I:* A solution of 3,3$^\prime$,3$^{\prime \prime }$-phosphanetriyltripropanoic acid (TCEP, $2\,\upmu$L, $1$ mM in water) was added to a solution of tssDNA (5$^\prime$-[thiol-C6]-TCGACTGTGTACGCGTGGGCGGTT-3$^\prime$, $500$ pmol) and ssDNA (5$^\prime$-AACCGCCCACGCGTACACAGTCGA-3$^\prime$, $500$ pmol) in buffer IV ($48\,\upmu$L). The resulting solution was homogenized and stirred for $16$ h at $37\,^{\circ }$C and $300$ rpm. The abbreviation [thiol-C6] stands for a 1-mercaptohex-6-yl group functioning as an anchor point for immobilization on gold interfaces as well as a spacer in between the corresponding gold interface and the DNA strands.

*ssDNA solution I:* ssDNA ($500$ pmol) was dissolved in buffer IV ($25\,\upmu$L).

*dsDNA solution II:* A solution of TCEP ($2\,\upmu$L, $1$ mM in water) was added to a solution of tssDNA ($500$ pmol) and ssDNA ($500$ pmol) in buffer III ($23\,\upmu$L). The resulting solution was homogenized, stirred for $16$ h at $37\,^{\circ }$C and $300$ rpm and degassed in an ultrasonic bath prior to use.

#### Nuclear protein lysates

For the preparation of nuclear protein lysates, the metastatic melanoma cell line Mel Im provided by Dr. Judith Johnson (LMU, Munich, Germany) was used. As described previously,^44,45^ cells were maintained in Dulbecco’s modified Eagle’s medium (DMEM) supplemented with penicillin ($$400$$ units$$\,\cdot$$ mL$$^{-1}$$), streptomycin ($$50$$ mg$$\,\cdot$$ mL$$^{-1}$$), and $$10\%$$ fetal calf serum (FCS; Sigma Aldrich, St. Louis, MO, USA). Cells were split at a ratio 1:5 on every third day and incubated at $$37\,^{\circ }$$C in a humidified atmosphere containing $$8\%$$ CO$${}_{2}$$. For preparation $$90\%$$ confluent T75 flasks of Mel Im cells were harvested. The isolation was performed according to the method of Dignam and colleages^46^. After harvesting, cell pellets were resuspended in $$400\,\upmu$$L buffer I ($${}^*$$) and incubated on ice for $$15$$ min. Then $$25\,\upmu$$L of the nonionic surfactant $$10\%$$ Nonident^®^-P-40 was added and gently mixed. To separate the nuclei, the samples were now centrifuged for $$1$$ min at $$10,000$$ rpm and $$4\,^{\circ }$$C. Afterwards, the supernatant containing the cytoplasmic fraction was decanted. For isolation of nuclear proteins, the nuclear pellet was resuspended in $$50\,\upmu$$L buffer II ($$^{**}$$) and shaked for $$15$$ min at $$4\,^{\circ }$$C followed by centrifugation for $$10$$ min at $$13,000$$ rpm and $$4\,^{\circ }$$C. The supernatant now containing the core proteins was transferred in fresh $$1.5$$ mL reaction vials and snap-frozen in liquid nitrogen. Afterwards, proteins were stored at $$-80\,^{\circ }$$C.

*Protein sample:* Half an aliquot of nuclear protein lysate was diluted with buffer III ($25\,\upmu$L) and stirred for $1$ h at $21\,^\circ$C and $300$ rpm in a nitrogen atmosphere.

*Protein blind sample:* Half an aliquot of nuclear protein lysate was diluted with dsDNA solution II ($25\,\upmu$L) and stirred for $1$ h at $21\,^\circ$C and $300$ rpm in a nitrogen atmosphere.

*EGR2 mRNA abundance:* The ratio of EGR2 mRNA to total mRNA in melanoma cells was determined as an average of six melanoma lines with two experiments each.

### Sensor preparation

**Figure 6:**
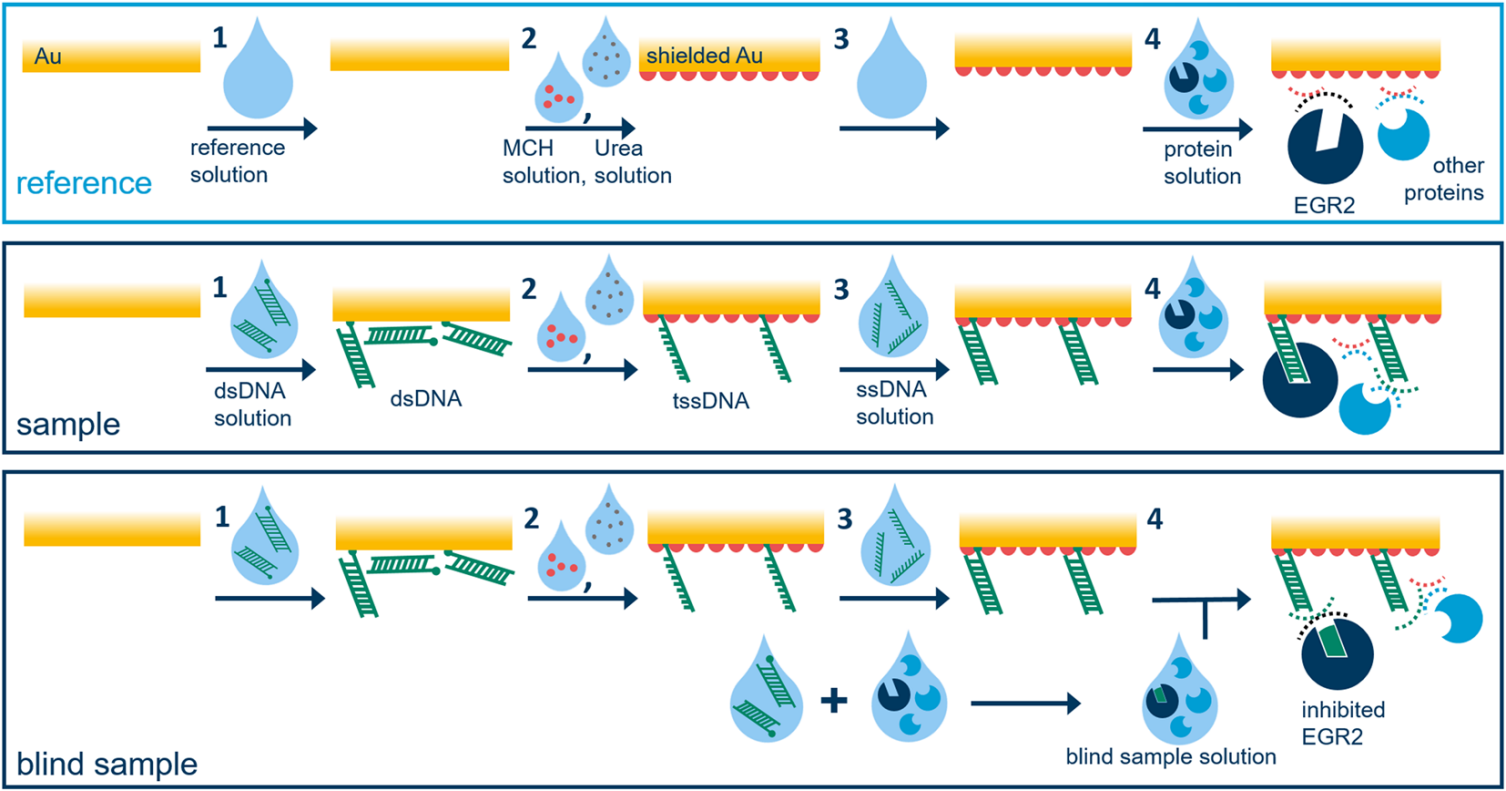
Illustration of most important sensor preparation procedures. **Top:** Reference measurement spots are covered either in water or buffer solution in the first and third step. In the second step they are treated with a MCH solution and Urea solution (not shown) resulting in a SAM which shields the gold surface thus preventing unspecific binding of nucleus proteins in these areas in the final step. **Center:** Sample measurement spots are treated with a dsDNA solution first and subsequently with MCH solution and Urea solution (not shown). The resulting biofilm consists of tssDNA and MCH. The immobilized tssDNA can be hybridized by treatment with the complementary ssDNA oligomer to form a dsDNA film or left denatured (not shown). If EGR2 transcription factors are present in the protein mixture, they can selectively bind to the immobilized DNA oligomers. **Bottom:** Blind sample measurement spots are prepared in the same manner as sample measurement spots are. The only difference is that the protein mixture is treated with a dsDNA solution $ex-situ$ beforehand, which inhibits the selective EGR2 immobilization on the sensor surface.

#### Sample sensor preparation for measurement of sample and reference query fields

The sensor was cleaned with O${}_{3}$, a solution of five parts H${}_{2}$SO${}_{4}$ ($96\%$) to one part H${}_{2}$O${}_{2}$ ($30\%$) and water as well as dried with N${}_{2}$ prior to the initial measurement. It was cleaned again with the acidic solution, water and dried with N${}_{2}$ after the initial measurement prior to the first functionalization step.

*Step 1:* The five water reference query fields were covered with water ($1.5\,\upmu$L each). The five buffer reference query fields were covered with buffer IV ($1.5\,\upmu$L each). The remaining 15 query fields were covered with dsDNA solution I ($1.5\,\upmu$L each). After leaving the sensor undisturbed for $1$ h at $21\,^\circ$C, it was rinsed thoroughly with water and dried with N${}_{2}$. The main binding events occurring in this step are visualized in Fig. 6 (reference case and sample case, step 1).

*Step 2:* All query fields were covered with a solution of 6-sulfanyl-1-hexanol (6-mercapto-1-hexanol, MCH, $1$ mM, $500\,\upmu$L). After $1$ h at $21\,^\circ$C the sensor chip was rinsed thoroughly with water, dried with N${}_{2}$ and covered with a solution of carbonyl diamide (Urea, saturated, $500\,\upmu$L). After $1$ h at $21\,^\circ$C the sensor chip was rinsed thoroughly with water and dried with N${}_{2}$. A simplified version of the microscopic situation at the active sensing area interface is displayed in Fig. 6 (reference case and sample case, step 2).

*Step 3:* The five water reference query fields were covered with water ($1.5\,\upmu$L each). The five buffer reference query fields and the five ssDNA query fields were covered with buffer IV ($1.5\,\upmu$L each). The ten dsDNA query fields were covered with ssDNA solution I ($1.5\,\upmu$L each). After leaving the sensor undisturbed for $1$ h at $21\,^\circ$C, it was rinsed carefully with water and dried with N${}_{2}$. The result of the hybridization process is depicted in Fig. 6 (sample case, step 3).

*Step 4:* All query fields were covered with protein sample solution ($50\,\upmu$L). After leaving the sensor undisturbed for $1$ h at $21\,^\circ$C, it was rinsed thoroughly with buffer III and water and dried with N${}_{2}$. A simplified version of the selective immobilization of EGR2 as well as the chemical inertness of the shielded interface against the nucleus proteins is visualized in Fig. 6 (reference case and sample case, step 4).

#### Blind sample sensor preparation for measurement of blind sample and reference query fields

The sensor was cleaned with O${}_{3}$, a solution of five parts H${}_{2}$SO${}_{4}$ ($96\%$) to one part H${}_{2}$O${}_{2}$ ($30\%$) and water as well as dried with N${}_{2}$ prior to the initial measurement. It was cleaned again with the acidic solution, water and dried with N${}_{2}$ after the initial measurement prior to the first functionalization step.

*Step 1:* The five water reference query fields were covered with water ($1.5\,\upmu$L each). The five buffer reference query fields were covered with buffer IV ($1.5\,\upmu$L each). The remaining 15 query fields were covered with dsDNA solution I ($1.5\,\upmu$L each). After leaving the sensor undisturbed for $1$ h at $21\,^\circ$C, it was rinsed thoroughly with water and dried with N${}_{2}$. The main binding events occurring in this step are visualized in Fig. 6 (reference case and blind sample case, step 1).

*Step 2:* All query fields were covered with a solution of MCH, $1$ mM, $500\,\upmu$L). After $1$ h at $21\,^\circ$C the sensor chip was rinsed thoroughly with water, dried with N${}_{2}$ and covered with a solution of carbonyl diamide (Urea, saturated, $500\,\upmu$L). After $1$ h at $21\,^\circ$C the sensor chip was rinsed thoroughly with water and dried with N${}_{2}$. A simplified version of the microscopic situation at the active sensing area interface is displayed in Fig. 6 (reference case and blind sample case, step 2).

*Step 3:* The five water reference query fields were covered with water ($1.5\,\upmu$L each). The five buffer reference query fields and the five ssDNA query fields were covered with buffer IV ($1.5\,\upmu$L each). The ten dsDNA query fields were covered with ssDNA solution I ($1.5\,\upmu$L each). After leaving the sensor undisturbed for $1$ h at $21\,^\circ$C, it was rinsed carefully with water and dried with N${}_{2}$. The result of the hybridization process is depicted in Fig. 6 (blind sample case, step 3).

*Step 4:* All query fields were covered with blind protein sample solution ($50\,\upmu$L). After leaving the sensor undisturbed for $1$ h at $21\,^\circ$C, it was rinsed thoroughly with buffer III and water and dried with N${}_{2}$. The chemical inertness of the interface against the nucleus proteins including the previously inhibited EGR2 is visualized in a simplified manner in Fig. 6 (reference case and blind sample case, step 4).

## Data Availability

The datasets generated during and/or analysed during the current study are available from the corresponding author on reasonable request.
